# Supplementary material for: Surgical Prehabilitation in Patients with Gastrointestinal Cancers: Impact of Unimodal and Multimodal Programs on Postoperative Outcomes and Prospects for New Therapeutic Strategies—A Systematic Review
Source: Cancers (Basel). 2023 Mar 21;15(6):1881. doi: 10.3390/cancers15061881 (PMC10047365; doi:10.3390/cancers15061881)
Supplement: Supplementary file 1 [file cancers-15-01881-s001.zip › cancers-2213572-supplementary.pdf]

Table S1. Main population characteristics

Studies	Country	Tumor stages and characteristics	Malnu-trition risk	Sex (♀)	Age (years)	BMI (kg/m²)	Neoadjuvant treatment and type of surgery	Surgical approaches	Operative time (mins)	Length of stay (days)
**Nutritional interventions**										
He<br>2022<br>n=67<br>[25]	CN	Stages II, III, IV<br>- Antrum 53%<br>- Gastric body 32%<br>- Esophagogastric junction 15%	86%[a]	29%	INT: 63.2±12.0<br>CO: 60.5±9.4	INT: 22.0±3.0<br>CO: 23.1±2.4	Gastrectomy:<br>- Distal subtotal 62%<br>- Total 33%<br>- Proximal subtotal 5%	-	INT: 207.0±33.1<br>CO: 203.3±41.1	-
Tesar<br>2022<br>n=120<br>[26]	CZ	Stages I, II, III<br>- Rectum 40%<br>- Right colon 36%<br>- Left colon 24%	29%[a]	34%	INT: 67.5±10.0<br>CO: 63.1±12.5	INT: 27.2±4.8<br>CO: 26.0±5.8	-	- Laparoscopy 76%<br>- Open 24%	-	INT: 10.9±9.0<br>CO: 11.2±8.0
Lee<br>2021<br>n=176<br>[27]	KOR	Stages I, II, III, IV<br>- Left colon 62%<br>- Right colon 38%	16%[a]	34%	INT: 65.3±9.2<br>CO: 65.3±11.7	INT: 24.4±3.5<br>CO: 24.1±4.1	- Anterior resection 60%<br>- Right hemicolectomy 33%<br>- Left hemicolectomy 5%<br>- Transverse colectomy 2%	- Laparoscopy 98%<br>- Conversion to open 2%	-	INT: 7.6±2.5<br>CO: 7.4±2.3
Okabayashi<br>2020<br>n=208<br>[28]	JP	Stages not mentioned<br>- Hepatocellular carcinoma 91%<br>- Intrahepatic cholangiocellular carcinoma 9%	-	31%	INT: 73.0±10.7<br>CO: 73.0±9.8	-	Hepatic resection:<br>- Subsegment 52%<br>- 1 segment 33%<br>- ≥2 segments 15%,	-	INT: 179[43-527]<br>CO: 167[57-519]	INT: 10[7-157]<br>CO: 12[5-144]
Ashida<br>2019<br>n=24<br>[29]	JP	Stages not mentioned<br>- Bile duct carcinoma 35%<br>- Ampullary carcinoma 20%<br>- Pancreatic carcinoma 15%<br>- Others 25%	-	45%	INT: 64.0±11.0<br>CO: 69.0±6.0	INT: 22.3±4.6<br>CO: 21.4±2.5	- Whipple 100%	- Open 100%	INT: 414.0±121.0<br>CO: 490.0±153.0	-
**Physical activity interventions**										
Berkel<br>2022<br>n=74<br>[30]	NL	Stages not mentioned<br>- Colon 95%,<br>- Rectum 5%	-[b]	47%	INT: 74.0±7.0<br>CO: 73.0±6.0	INT: 29.8±4.1<br>CO: 30.5±4.9	NAT: 5%<br>- Right hemicolectomy 47%<br>- Sigmoid colectomy 32%	- Laparoscopy 77%<br>- Conversion to open 19%<br>- Open 4%	-	INT: 8.4±7.4<br>CO: 9.1±7.0

							- Left hemicolectomy 9%<br>- Transverse hemicolectomy 3%<br>- Abdominal perineal resection 2%<br>- Low anterior resection 2%<br>- Subtotal colectomy 2%<br>- Other 3%			
Steffens<br>2021<br>n=22<br>[31]	AUS	Stages not mentioned<br>- Primary rectal 14%<br>- Recurrent rectal 32%<br>- Appendix adenocarcinoma 27%<br>- Pseudomyxoma peritonei 14%<br>- Colorectal 9%<br>- Other 4%	-	46%	INT: 62[48-72]<br>CO: 66[46-70]	-	NAT: 17%<br>- Pelvic exenteration 50%<br>- Cytoreductive surgery & hyperthermic intraperitoneal chemotherapy 50%	- Open 100%	-	INT: 36[15-52]<br>CO: 29[15-53]
Karlsson<br>2019<br>n=23<br>[32]	SE	Stages I, II, III, IV<br>- Colon 86%<br>- Rectum 14%	-	62%	INT: 83[76-85]<br>CO: 74[73-76]	-	NAT: 14%<br>No information on type of surgery	- Laparoscopy 71%<br>- Open 29%	INT:<br>colon 150[140-164]<br>rectum 401[-]<br>CO:<br>colon 159[145-176]<br>rectum 529[441-616]	INT: 5[4-6]<br>CO: 6[4-7]
**Probiotics and symbiotics interventions**										
Roussel<br>2022<br>n=54<br>[33]	FR	Stages not mentioned<br>- Hepatocellular carcinoma 100%	-	15%	INT: 66.0±8.9<br>CO: 66.9±6.7	-	NAT: 11%<br>- Minor liver resection 93%<br>- Major liver resection 7%	-	INT: 130[40-400]<br>CO: 100[40-290]	-
Polakowski<br>2019<br>n=120<br>[34]	BR	Stages I, II, III	-	47%	INT: 60.9±6.7<br>CO: 58.9±6.3	INT: 24.8±3.2<br>CO: 24.9±2.9	NAT: 0%<br>- Abdominal rectosigmoidectomy 49%<br>- Rectosigmoidectomy with colostomy /ileostomy 44%<br>- Right colectomy 7%	-	-	INT: 3.0±1.0<br>CO: 4.0±18.0
**Nutritional and physical activity interventions**										
Ausania<br>2019<br>n=40	ES	Stages not mentioned<br>- Ductal carcinoma 45%	INT: 100%[c]<br>CO: -	45%	INT: 66[38-80]<br>CO: 66[38-81]	INT: 24.8[-]<br>CO: 26.5[-]	- Whipple 100%	- Open 100%	-	INT: 11[7-46]<br>CO: 13[7-60]

[35]		- Ampullary carcinoma 40%<br>- Cholangiocarcinoma 7.5%<br>- Intraductal papillary mucinous neoplasm carcinoma 7.5%								
Minnella 2018<br>n=68<br>[36]	CA	Stages I, II, III<br>- Esophagus 80%,<br>- Gastric 20%	16%[a]	25%	INT: 67.3±7.4<br>CO: 68.0±11.6	INT: 26.1±4.8<br>CO: 25.7±4.7	NAT: 69%<br>- Esophagectomy 80%<br>- Partial gastrectomy 12%<br>- Total gastrectomy 8%	- Open 57%<br>- Laparoscopy 43%	INT: 195[170-226]<br>CO: 226[179-315]	INT: 8[6-12]<br>CO: 7[6-13]
**Multimodal interventions**										
Allen 2022<br>n=54<br>[37]	UK	Stages I, II, III, IV	-	15%	INT: 65.0±6.0<br>CO: 62.0±9.0	INT: 28.1±4.8<br>CO: 27.7±5.1	NAT: 100%<br>- Open esophagectomy 84%<br>- Total gastrectomy 11%<br>- Resection abandoned 5%	-	INT: 515[429-528]<br>CO: 475[431-536]	INT: 11[9-21]<br>CO: 16[9-33]
Carli 2020<br>n=120<br>[38]	CA	Stages I, II, III, IV<br>- Colon 72%<br>- Rectum 28%	64%[d]	53%	INT1: 78[72-82]<br>INT2: 82[74-84]	INT1: 25[23-30]<br>INT2: 26[24-31]	NAT: 12%<br>- Right hemicolectomy 42%<br>- Low anterior resection 15%<br>- Anterior/sigmoid resection 14%<br>- Left hemicolectomy 12%<br>- Abdominoperineal resection 7%<br>- Ileocecal resection 3%<br>- Subtotal colectomy 3%<br>- Transverse colectomy 2%<br>- Other 2%	- Laparoscopy 79%<br>- Open 21%	INT1: 180[110-280]<br>INT2: 180[130-300]	INT1: 4[3-8]<br>INT2: 5[3-9]
Minnella 2020<br>n=42<br>[39]	CA	Stages I, II, III<br>- Colon 74%,<br>- Rectum 26%	41%[c]	38%	INT1: 67[60-72]<br>INT2: 67[50-76]	INT1: 29[23-32]<br>INT2: 26[23-34]	NAT: 19%<br>No information type of surgery	- Laparoscopy 100%	INT1: 183.5±51.4<br>INT2: 216.7±52.4	INT1: 4[3-6]<br>INT2: 4[3-5]

Results are presented as mean±SD, median[IQR]; [a] Nutritional Risk Screening (NRS), [b] Short Nutritional Appetite Questionnaire (SNAQ), [c] Patient-Generated Subjective Global Assessment (PG-SGA), [d] Subjective Global Assessment (SGA)

Abbreviations: -: no data, BMI: Body mass index, CN: China, CZ: Czech Republic, KOR: South Korea, JP: Japan, NL: Netherlands, AUS: Australia, SE: Sweden, FR: France, BR: Brazil, ES: Spain, CA: Canada, UK: United Kingdom, INT: Intervention group, CO: Control group, INT1: Intervention N°1, INT2: Intervention N°2, NAT: Neoadjuvant treatment
